# The role of RNA structure in translational regulation by L7Ae protein in archaea

**DOI:** 10.1261/rna.068510.118

**Published:** 2019-01

**Authors:** Lin Huang, Saira Ashraf, David M.J. Lilley

**Affiliations:** Cancer Research UK Nucleic Acid Structure Research Group, The University of Dundee, Dundee DD1 5EH, United Kingdom

**Keywords:** gene regulation, RNA structure, kink-turn, X-ray crystallography

## Abstract

A recent study has shown that archaeal L7Ae binds to a putative k-turn structure in the 5′-leader of the mRNA of its structural gene to regulate translation. To function as a regulator, the RNA should be unstructured in the absence of protein, but it should adopt a k-turn-containing stem–loop on binding L7Ae. Sequence analysis of UTR sequences indicates that their k-turn elements will be unable to fold in the absence of L7Ae, and we have demonstrated this experimentally in solution using FRET for the *Archaeoglobus fulgidus* sequence. We have solved the X-ray crystal structure of the complex of the *A. fulgidus* RNA bound to its cognate L7Ae protein. The RNA adopts a standard k-turn conformation that is specifically recognized by the L7Ae protein, so stabilizing the stem–loop. In-line probing of the natural-sequence UTR shows that the RNA is unstructured in the absence of L7Ae binding, but folds on binding the protein such that the ribosome binding site is occluded. Thus, L7Ae regulates its own translation by switching the conformation of the RNA to alter accessibility.

## INTRODUCTION

Riboregulation takes many forms. It can be mediated by small RNA molecules (noncoding regulatory RNA), metabolites (in the riboswitches) or proteins. A number of ribosomal proteins are subject to autoregulation; a good example is the binding of bacterial S15 protein to a specific structure within the 5′ region of its mRNA to repress translation (Serganov et al. 2003). L7Ae is an archaeal ribosomal protein that has attracted recent interest in terms of autoregulation (Daume et al. 2017). This is a member of a family of structure-selective RNA binding proteins that includes yeast L30e and the human 15.5K protein (Koonin et al. 1994; Watkins et al. 2000). These proteins fulfill a number of roles; they are important components in the large ribosomal subunit (Ban et al. 2000), box C/D (Kuhn et al. 2002; Szewczak et al. 2002; Watkins et al. 2002), and H/ACA (Rozhdestvensky et al. 2003; Hamma and Ferré-D'Amaré 2004; Li and Ye 2006) snoRNPs and U4 snRNA in the spliceosome cycle (Nottrott et al. 1999; Vidovic et al. 2000). In addition, they are components of U3 snoRNP (Marmier-Gourrier et al. 2003), telomerase (Pogacić et al. 2000), and RNaseP that is involved in tRNA 5′-end processing (Cho et al. 2010). L7Ae-family proteins are thus essential in many cellular processes including ribosome structure, spliceosome assembly and guided site-specific modification of RNA.

The L7Ae family of proteins selectively bind to a widespread structural motif in RNA called the kink turn (k-turn) (Klein et al. 2001), which has recently been reviewed in depth (Huang and Lilley 2018). The k-turn is a motif found in duplex RNA comprising a short (usually 3 nt) bulge followed by tandem *trans*-sugar-Hoogsteen G:A base pairs. The RNA can adopt one of two conformations; either a relatively extended structure similar to a typical 3-nt bulge, or a tightly kinked structure where the helical axis includes an angle of 50° (Goody et al. 2004). The kinked conformation requires juxtaposition of the two minor grooves, with formation of key cross-strand hydrogen bonds at the interface (Lescoute et al. 2005; Liu and Lilley 2007; Daldrop and Lilley 2013). The major groove is splayed open on the outer face of the RNA. The kinked structure of the k-turn requires stabilization that can be effected by a number of alternative processes. Some, but importantly not all, k-turns become stabilized on addition of metal ions (Goody et al. 2004; Liu and Lilley 2007). Whether or not a given k-turn folds in response to metal ions depends upon its sequence, and we have found that two sequence elements act as key determinants of this behavior (McPhee et al. 2014; Ashraf et al. 2017). Protein binding provides an alternative way to stabilize the folded k-turn. The great majority of k-turns can be stabilized by the binding of L7Ae-family proteins (Turner et al. 2005), which bind with very high affinity (Turner and Lilley 2008). Importantly, even those k-turns that do not fold on addition of metal ions generally fold on binding L7Ae (McPhee et al. 2014) or 15.5K (Huang et al. 2017) proteins. Examples of k-turns that exhibit this behavior are the archaeal box C/D k-turns (Ashraf et al. 2017) and the human U4 snRNA k-turn (McPhee et al. 2014).

In a recent study, Randau and colleagues (Daume et al. 2017) obtained evidence pointing to an interesting new mechanism of genetic regulation in archaea, involving the interaction between L7Ae and the transcript of its structural gene. Using the archaeon *Sulfolobus acidocaldarius*, they performed a RIP-Seq analysis to provide the L7Ae-RNA interactome, and so identified over 100 RNA species that bind L7Ae in the cell, of which 59 were box C/D snoRNAs. This also included 32 mRNA species, one of which was the transcript of *l7ae*, the structural gene encoding L7Ae protein. Detailed analysis of the data indicated that the principal site of binding lay in the 5′-untranslated region (UTR) of the transcript, the sequence of which was consistent with the formation of a short stem–loop structure that contained a potential k-turn structure. The stem–loop overlapped with the ribosome binding site, suggesting a potential mechanism for translational regulation, and this was supported by reporter assays showing the down-regulation of translational in the presence of L7Ae. Daume et al. (2017) also found that potential k-turn-forming hairpin structures were a conserved feature in the 5′-UTR regions of archaeal genes encoding L7Ae.

This suggests a mechanism of gene regulation in which the RNA structure of the transcript plays a key role. It seems probable that the 5′-UTR sequence would be unstructured and accessible to the translation machinery until bound by L7Ae. Thereupon the k-turn would fold to stabilize the stem–loop that would occlude the ribosome binding site. In this work we have sought to test two structural aspects of this model. First, in the absence of L7Ae the RNA should not fold into the kinked conformation k-turn, but rather should behave like the archaeal box C/D k-turn that is unfolded in the presence of metal ions. Second, when present L7Ae should bind so that the k-turn folds and a standard L7Ae-k-turn complex is formed. We have found that both of these criteria are met for the *Archaeoglobus fulgidus l7ae* leader RNA, supporting the proposed regulatory mechanism.

## RESULTS

### The predicted folding ability of k-turns in L7Ae gene UTRs

**FIGURE 1.**
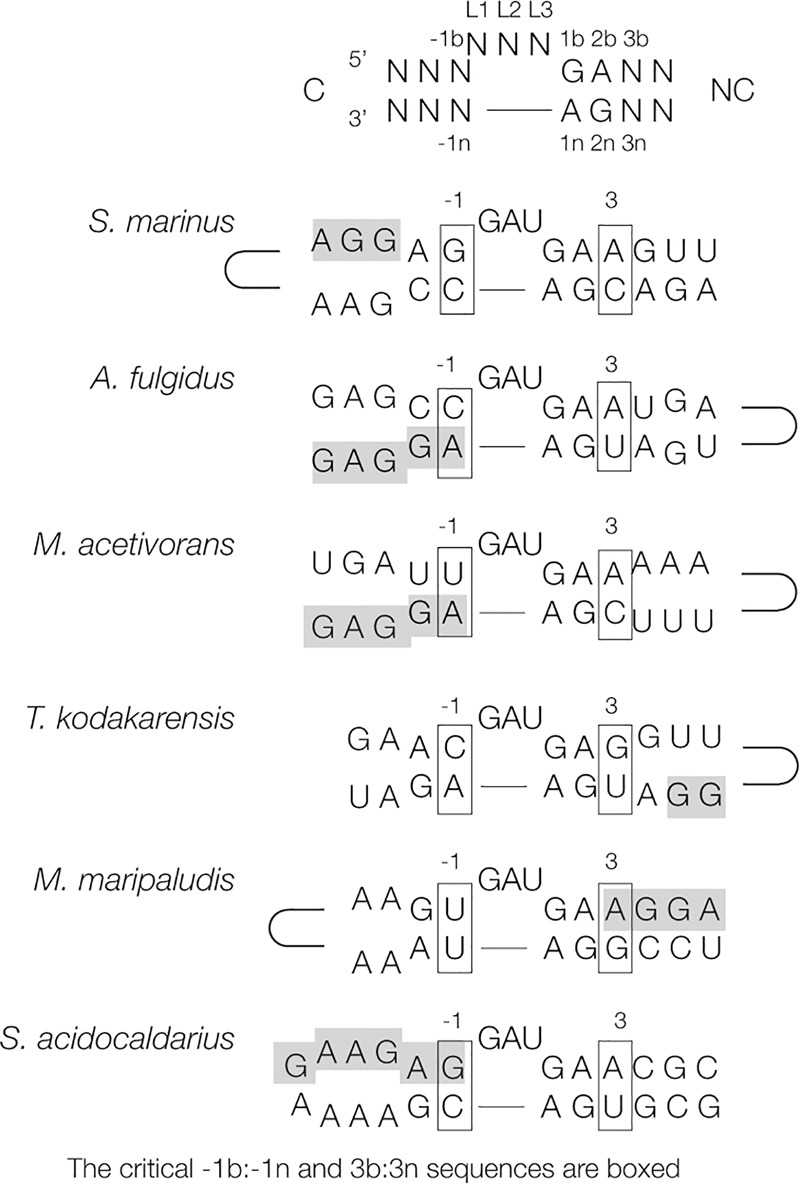
Potential k-turn sequences occurring in the 5′-UTR sequences of the mRNA of archaeal *l7ae* genes. Each sequence is drawn in the form of a hairpin loop containing a putative k-turn structure, with the critical −1b:−1n and 3b:3n sequences boxed. The dependence of ion-dependent folding on the 3b:3n sequence is summarized in Supplemental Figure S1. The Shine–Dalgarno (ribosome binding site) sequences are shaded. The locations of the terminal loops are indicated schematically. The nomenclature of sequence positions for a standard k-turn is shown at the *top*. Sequence information was taken from Daume et al. (2017).

The 5′-UTR regions of the *l7ae* genes from six different archaeal species (first identified by Daume et al. 2017) are drawn in Figure 1 to show how each could potentially form a hairpin loop containing a k-turn structure. Each sequence can be drawn as a stem–loop containing a GAU bulge followed by tandem G:A and A:G base pairs. The nomenclature of sequence positions in the k-turn (Liu and Lilley 2007) is shown at the top of Figure 1. The polarity of the k-turn within the stem–loop is evenly divided such that three have the terminal loop on the C-helix and three on the NC-helix. In each case, the Shine–Dalgarno ribosome binding site (shaded in Fig. 1) is in very close proximity to the k-turn motif, although its position with respect to the k-turn varies from sequence to sequence.

In general, these stem–loop structures appear to be of low intrinsic stability, especially at the elevated temperatures at which many of these thermophilic organisms live. In all cases, the C-helix has a limited number of Watson–Crick base pairs, and some of the sequences have few Watson–Crick base pairs following the G:A base pairs. If the stem–loop structures do form, we can ask how readily might the k-turn fold into its tightly kinked conformation in the absence of protein binding. We have previously studied this in some depth (McPhee et al. 2014; Ashraf et al. 2017), and have identified two critical sequence elements that determine the ability of a k-turn to fold in response to the presence of metal ions.

1. The 3b:3n sequence. We showed that the 3b:3n sequence that follows the tandem G:A and A:G base pairs in the well-characterized standard k-turn Kt-7 has a major influence on its ion-induced folding (McPhee et al. 2014). We noted that Watson–Crick base pairs prevent folding, while 3b = C or 3n = G conferred ion-induced folding. We have previously presented crystallographic evidence for the origin of the 3n = G rule, showing that the guanine O6 atom is directly bound to a magnesium ion in the major groove (McPhee et al. 2014). For ease of reference, the sequence-dependent data are summarized in Supplemental Figure S1.
2. The −1b:−1n sequence. Recently we have found that the −1b:−1n sequence that precedes the bulge can have a strong influence on ion-induced folding (Ashraf et al. 2017). Systematic comparison of the folding of Kt-7 and the box C/D k-turns showed that −1b:−1n = C:G confers folding in metal ions, while inversion to −1b:−1n = G:C prevents folding under the same conditions. The great majority of natural k-turns have −1b:−1n = C:G, and −1b:−1n = G:C is quite rare. No systematic study has been made of the folding properties resulting from all possible sequences at the −1b:−1n position, but we have found that −1b:−1n = C:A prevents ion-induced folding of Kt-7 (Supplemental Fig. S2).

These rules appear to apply generally for all k-turns studied. Importantly, every k-turn that has been analyzed, irrespective of the 3b:3n or −1b:−1n sequences, undergoes folding into the kinked conformation on addition of L7Ae protein (McPhee et al. 2014; Ashraf et al. 2017).

Insofar as possible we have applied the above rules to the *l7ae* URT k-turn sequences. With a single exception (*M. maripaludis*), none of the elements has 3b = C or 3n = G, and with that exception none is expected to fold well in response to metal ions. In addition, none of the six sequences has −1b:−1n = C:G, and two have G:C and one has C:A. The remaining three have −1b:−1n sequences whose folding properties have not been studied. Taken together, it is probable that all the sequences are unable to fold into a stable k-turn conformation in response to the addition of metal ions.

### Analysis of the folding of the *l7ae* gene k-turn from *Archaeoglobus fulgidus*

If a k-turn is to behave as a translational switch to regulate L7Ae protein synthesis, it would require two characteristics. It should not fold into a stable k-turn conformation in the absence of the protein, despite the presence of metal ions, but it should bind L7Ae protein and thereupon fold into the kinked conformation. We chose the *A. fulgidus l7ae* UTR to explore this question. It has 3b:3n = A:U, and −1b:−1n = C:A, both of which prevent ion-induced folding in Kt-7 (Supplemental Fig. S2; McPhee et al. 2014).

**FIGURE 2.**
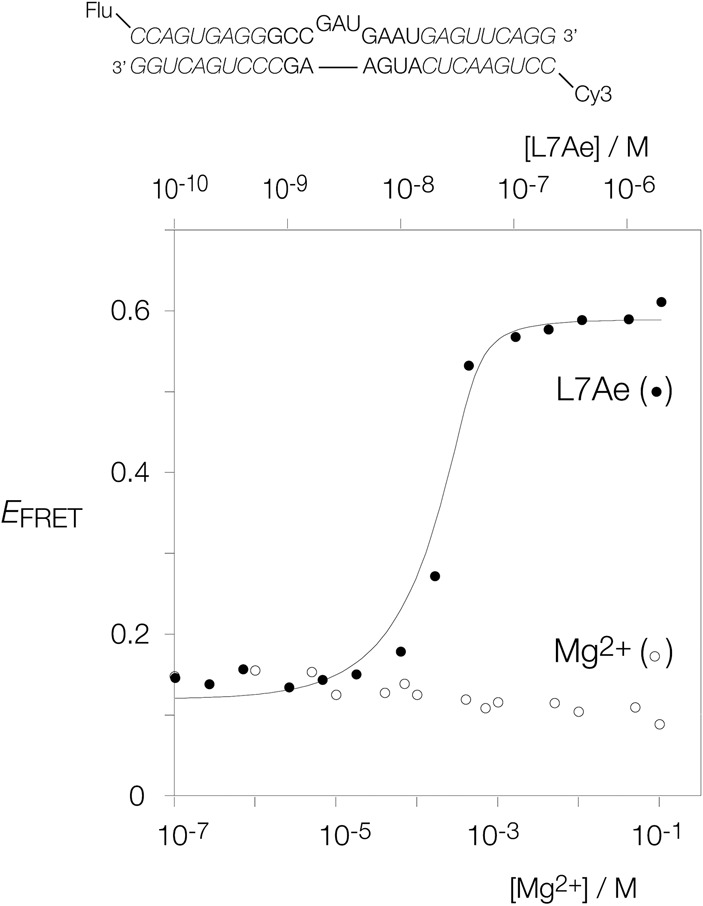
Folding of the *A. fulgidus l7ae* 5′-UTR k-turn in response to Mg^2+^ ions and L7Ae studied by FRET. The RNA shown at the *top* was synthesized with 5′ fluorescein donor and Cy-3 acceptor. The nucleotides written in italic extend the *A. fulgidus* sequence to make a duplex suitable for the FRET experiments. A solution of 20 nM fluorophore-labeled RNA was titrated with either Mg^2+^ ions (*lower* concentration scale) or L7Ae protein (*upper* concentration scale) and *E*_FRET_ measured in the steady state. *E*_FRET_ is plotted as a function of Mg^2+^ ions (open circles) and L7Ae protein (closed circles). The RNA clearly fails to fold in response to the addition of the metal ions. However, addition of L7Ae results in a structural transition, and the data have been fitted (line) to Equation 1.

We studied the folding of this element using fluorescence resonance energy transfer (FRET). For this purpose we synthesized a dsRNA containing the central k-turn, extending the natural sequence to have C and NC arms of 12 and 13 bp, respectively (Fig. 2, top). The 5′ termini were labeled with fluorescein donor and Cy3 acceptor fluorophores. Fluorescence emission spectra were recorded in the steady state, and FRET efficiency (*E*_FRET_) calculated using the acceptor normalization method (Clegg 1992). If the RNA is kinked by the folding of the k-turn, the separation between the fluorophores will decrease and consequently *E*_FRET_ will increase.

The fluorescent RNA was titrated with either Mg^2+^ ions or *A. fulgidus* L7Ae protein (*Af*L7Ae), and *E*_FRET_ has been plotted as a function of ion or protein concentration (Fig. 2). It is immediately apparent that the two sets of data are quite different in character. The value of *E*_FRET_ as a function of Mg^2+^ concentration (open circles) remains at a low value (<0.18) across the whole range of ionic concentration. In contrast, addition of L7Ae protein (closed circles) results in a marked increase in *E*_FRET_ at stoichiometric concentration, reaching a plateau value of 0.6. This is the standard behavior of a k-turn that folds on binding a member of the L7Ae family of proteins (Turner et al. 2005; Turner and Lilley 2008; Huang et al. 2017). Thus, the *A. fulgidus l7ae* UTR folds in a manner consistent with the formation of a normal k-turn in response to the binding of L7Ae, but is unable to fold stably on addition of metal ions alone.

### Crystal structure of *A. fulgidus* L7Ae bound to its cognate UTR k-turn

**FIGURE 3.**
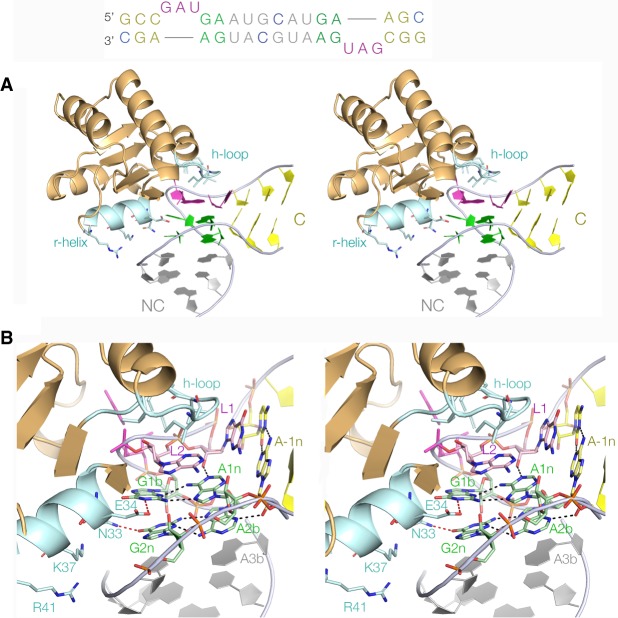
The structure of the *A. fulgidus* L7Ae–RNA complex. X-ray crystal structure of *Af*L7Ae bound to the k-turn of the *l7ae* 5′-UTR k-turn. The RNA (sequence shown at *top*) contains two copies of the potential k-turn related by twofold rotational symmetry. The two nucleotide changes were made to create full base-pairing at −3n and 5n; these are highlighted in blue. Our standard color-coding has been used for the k-turn, with the G:A base pairs green, the C and NC helices gray and yellow, respectively, and the loop magenta. This color scheme is also used in the structural images. The molecular graphics are shown as parallel-eye stereoscopic images. (*A*) The overall structure of the complex. The L7Ae protein is shown in cartoon form colored orange apart from the recognition helix and hydrophobic loop that are highlighted in cyan. Important amino acid side chains are shown in stick form. (*B*) The protein–RNA interactions in greater detail. The recognition helix (*r-helix*) is located in the widened major groove on the outer face of the k-turn, and the side chains of E34 and N33 are hydrogen bonded to the nucleobases of G1b and G2n, respectively. The hydrophobic loop (*h-loop*) sits over the L2, L1 region of the k-turn.

The change in *E*_FRET_ on binding *Af*L7Ae to the *A. fulgidus l7ae* UTR is consistent with the formation of a folded k-turn at the binding site. This was explored further by X-ray crystallography. We synthesized the RNA shown in Figure 3 (top) that comprises a twofold symmetrical duplex containing two k-turns. This required just two sequence changes from the natural UTR sequence, in the −3n and 5n positions (highlighted in blue). The RNA was co-crystallized with *Af*L7Ae, and the structure was solved at a resolution of 3.09 Å by molecular replacement using the structure of *Af*L7Ae bound to Kt-7 (PDB ID 4BW0) (Huang and Lilley 2013).

**FIGURE 4.**
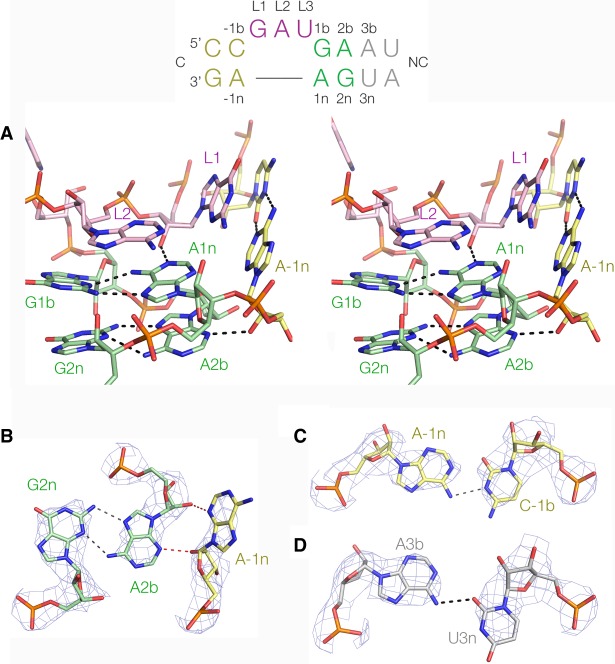
The structure of the *A. fulgidus* 5′-UTR k-turn in complex with L7Ae. The sequence and nucleotide nomenclature for the k-turn is shown at the *top*. (*A*) A parallel-eye stereoscopic image of the core of the k-turn. The RNA adopts the conformation of a standard, simple k-turn with two *trans*-sugar-Hoogsteen G:A base pairs and cross-strand hydrogen bonds from the O2′ groups of L1 and −1n ribose to the nucleobases of A1n and A2b, respectively. *B* through *D* show details from the structure with electron density from the 2F_o_−F_c_ map contoured at 2.5 σ. (*B*) The A2b:G2n base pair and its interaction with A-1n. A-1n N3 accepts a hydrogen bond from A2b O2′, and its O2′ donates one to A2b N3. The A2b:G2n base pair is associated by two hydrogen bonds. This k-turn is therefore a standard N3 structure (Daldrop and Lilley 2013). (*C*) The C-1b:A-1n base pair. This forms a *cis* base pair associated by a single hydrogen bond from A-1n N6 to C-1b N3. (*D*) The A3b:U3n base pair. This forms a *trans* base pair associated by a single hydrogen bond from A3b N6 to U3n O2.

In the crystal lattice, three two-k-turn units adopted a triangular arrangement, with six L7Ae molecules bound to six k-turns (Supplemental Fig. S3). The structure of a single k-turn bound to one molecule of *Af*L7Ae is shown in Figure 3 and the RNA structure is shown in detail in Figure 4. The overall structure of the complex (Fig. 3A) is closely similar to that of other L7Ae complexes, such as those with Kt-7 (Huang and Lilley 2013), box C/D (Moore et al. 2004), and U4 (Vidovic et al. 2000) k-turns. L7Ae places its recognition α-helix in the widened major groove that is splayed around the outside of the k-turn, and places a hydrophobic loop over the L2 nucleobase (Fig. 3B). The recognition helix makes the usual nonspecific interactions with the RNA (using the sidechains of K37 and R41) and specific hydrogen-bonding interactions with the G1b and G2n nucleobases of the tandem G:A base pairs (using E34 and N33, respectively). In addition, the O6 atom of G1b is located on the axis of the α-helix where its partial negative charge makes an electrostatic interaction with the positive pole of the helix dipole moment.

In the complex, the RNA is folded into a standard k-turn conformation (Fig. 4). L1 is stacked on the −1b:−1n base pair, and L2 adopts a *syn* conformation to stack on the 1b:1n G:A base pair (Fig. 4A). Both G:A base pairs are *trans*-sugar-Hoogsteen pairs, and the adenine nucleobases accept the usual cross-strand hydrogen bonds. L1 O2′ donates a hydrogen bond to A1n N1, while A-1n O2′ donates one to A2b N3 (Fig. 4B). Thus the k-turn adopts the N3 conformation (Daldrop and Lilley 2013), and the A2b:G2n base pair is associated by two hydrogen bonds. Parenthetically, this provides experimental confirmation of our previous prediction that k-turns with 3b:3n = A:U would adopt the N3 conformation (see Supplemental Fig. S1; Huang et al. 2016).

The *l7ae* UTR k-turn contains some sequence elements not previously present in k-turn structures. The A-1b:C-1n forms a *cis* base pair associated by a single hydrogen bond from A-1n N6 to C-1b N2 (Fig. 4C). The A:U base pair at the 3b:3n position is not a standard Watson–Crick pair; rather it adopts a *trans* conformation with a single hydrogen bond from A3b N6 to U3n O2 (Fig. 4D). The 4b:4n base pair is a standard *cis*-Watson–Crick pair.

### L7Ae-induced structural change in the *A. fulgidus l7ae* 5′-UTR stem–loop

**FIGURE 5.**
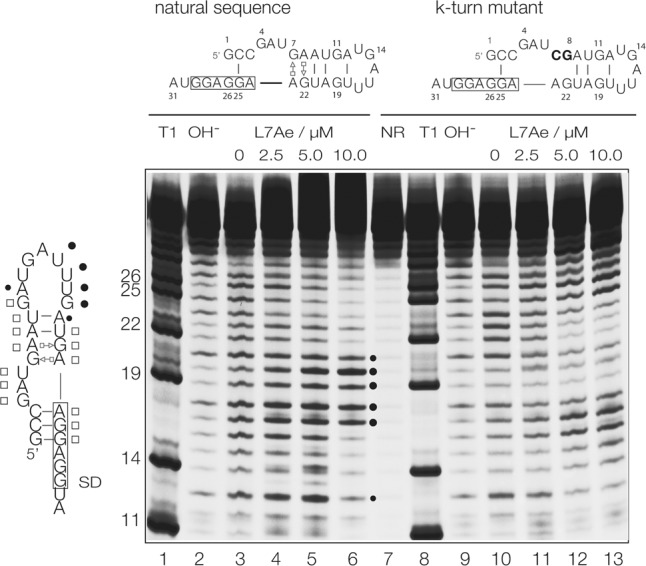
Analysis of conformational change in the *A. fulgidus* 5′-UTR RNA induced by the binding of *Af*L7Ae protein using in-line probing (Soukup and Breaker 1999). The sequences of the two RNA species studied are shown at the *top*, drawn in their potential stem–loop conformations. The boxed regions are the ribosome binding site. The mutant sequence (*right*) has two sequence changes (highlighted in bold) in the G:A base pairs that will prevent k-turn formation. Tracks 1 and 8 contain RNA cleaved by T1 nuclease that cuts 3′ to G nucleotides to provide a reference frame (sequence positions shown on the *left*). Tracks 2 and 9 contain natural-sequence RNA subject to hydroxide cleavage to generate cleavage through the RNA. Track 7 contains RNA not subjected to any degradation procedure, i.e., full-length RNA. Tracks 3 through 6 and 10 through 13 contain the results of in-line probing in the presence of 0, 2.5, 5, and 10 µM *Af*L7Ae for natural and mutant RNA, respectively. The black circles drawn on the fluorogram denote positions of reactivity in the natural 5′-UTR RNA in the presence of L7Ae. The schematic on the *left* shows the sequence of the natural 5′-UTR drawn in its stem–loop conformation. Filled circles denote positions of reactivity and open squares denote positions of protection, both in the presence of L7Ae.

In addition to spectroscopic and crystallographic studies, we have investigated the structural change in the k-turn-carrying stem–loop structure formed by the *A. fulgidus l7ae* 5′-UTR stem–loop RNA using chemical probing. For this experiment, the natural sequence of the 5′-UTR was used without any changes, and the results compared with those from a modified sequence in which the 1b and 2b nt were exchanged for C and G respectively to prevent adoption of the k-turn conformation. These were studied by in-line probing (Soukup and Breaker 1999) as a function of added L7Ae concentration. 5′-Cy5-labeled RNA was incubated at room temperature in 50 mM Tris-HCl (pH 8.3), 20 mM MgCl_2_, and 100 mM KCl for 40 h. Under these conditions, each phosphodiester linkage may be cleaved as a result of nucleophilic attack by its adjacent 2′-hydroxyl group if it can adopt the required in-line geometry. The products of cleavage are separated by electrophoresis in a denaturing polyacrylamide gel. In general, the extent of cleavage at a given position (i.e., the intensity of the product bands) is a measure of the flexibility of the RNA at that point, allowing it to sample the in-line conformation. The results are shown in Figure 5, with the regions of reactivity and protection shown on the schematic (left).

In the absence of L7Ae protein, both wild-type and mutant RNAs exhibited uniform cleavage along their length, consistent with a largely single-stranded character. However, on addition of *A. fulgidus* L7Ae to the unmodified UTR sequence, the section corresponding to the stem regions became protected, and that corresponding to the loop exhibited enhanced reactivity. In contrast, the mutant sequence gave a rather different pattern of bands indicative of a different mode of binding and not consistent with the same stem–loop formation. We know from earlier work that at higher L7Ae concentration nonspecific binding can occur, that does not result in the standard k-turn conformation. From a functional point of view it is apparent that the ribosome binding site is reactive (i.e., flexible) in the mutant, but becomes protected in the L7Ae complex for the natural sequence. These results are consistent with the formation of the stem–loop structure in the natural 5′-UTR that is stabilized by binding of L7Ae to the k-turn structure, with a resulting occlusion of the ribosome binding site.

## DISCUSSION

**FIGURE 6.**
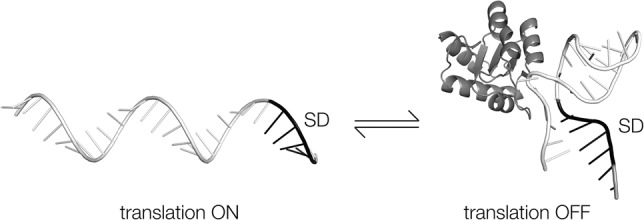
Cartoon showing the probable mechanism of regulation of translation by the binding of L7Ae to the 5′-UTR leader sequence. In the absence of the protein, the RNA is unstructured and the Shine–Dalgarno sequence (SD, black) is accessible, allowing translation to initiate. On binding L7Ae the stem–loop forms, stabilized by protein-k-turn binding. The Shine–Dalgarno sequence is now incorporated into the C-helix of the k-turn and thus occluded so that translation cannot be initiated.

We have shown that *A. fulgidus* L7Ae binds to the 5′-UTR of the mRNA of its structural gene, stabilizing the standard k-turn conformation that should occlude the ribosome binding site. In the absence of L7Ae protein, the stem–loop structure should be very unstable, as we demonstrate by in-line probing. This will be accentuated at the elevated temperature at which this thermophilic archaeon lives. Under these conditions, the equilibrium between unstructured RNA and the stem–loop will be heavily biased toward the open structure. Previous single-molecule studies from our laboratory are consistent with protein stabilization of k-turn structure occurring by conformational selection (Wang et al. 2012), and our FRET studies show that the k-turn is largely unfolded in the absence of L7Ae protein. We conclude that in the absence of protein the RNA is predominantly single-stranded, and it requires binding of L7Ae to the k-turn structure to stabilize the stem–loop structure. Until this happens the RNA should be available to ribosome binding and thus the RNA is translated. On binding L7Ae, the stem–loop becomes stable and the ribosome binding site occluded. These are exactly the characteristics required for a translational OFF switch that would self-regulate synthesis of the L7Ae protein, shown in cartoon form in Figure 6.

Daume et al. (2017) showed that elements that could potentially form k-turn structures exist in the 5′-UTRs of *l7ae* genes of a wide variety of archaea, and used reporter constructs to demonstrate regulation of translation in that from *S. acidocaldarius*. Application of sequence rules indicates the majority (probably all) will be unable to form k-turn structures in the absence of protein, so k-turn formation unaided by L7Ae binding cannot stabilize the stem–loop structure and translation can initiate until the k-turn elements are bound and folded by L7Ae. These elements were identified in both Crenarchaeota (e.g., *S. acidocaldarius*) and Euryarchaeota (e.g., *A. fulgidus*) that diverged very early in the evolution of life (>3 Gy ago) (Battistuzzi et al. 2004), so this regulatory mechanism is likely to be ancient. This extends further, into the eukaryota. In budding yeast, the 5′-UTR of the structural gene for L30e, another member of the L7Ae family, has a k-turn structure (Mao et al. 1999; Chao and Williamson 2004). This too has been shown to be involved in the self-regulation of L30e translation (Dabeva and Warner 1993). In addition, the motif incorporates a splice site, and evidence suggests that it is involved in the regulation of splicing (Eng and Warner 1991). It is therefore probable that the alteration of RNA structure consequent to the binding of L30e prevents the interaction with both the ribosome and U1 snRNA species.

Returning to the archaea, L7Ae has been shown to have multiple targets for regulation. In their RIP-Seq analysis of *S. acidocaldarius*, Daume et al. (2017) found that L7Ae also lowered translation of the structural gene for NOP5, another protein involved in the assembly of the box C/D snoRNP. In *T. kodakarensis*, a putative k-turn has been found in the mRNA of a bicistronic gene encoding the box C/D proteins NOP5 and fibrillarin (Jäger et al. 2014). These authors also identified a putative k-turn in the *cbf5* gene that encodes the pseudouridinylase enzyme of the H/ACA snoRNP complex (Jäger et al. 2014). Thus, the assembly of archaeal snoRNP species is likely to be regulated by L7Ae at multiple points. We have also recently shown that a subset of human box C/D snoRNPs are subject to probable regulation by N^6^-methylation of adenine within the core of the k-turn structure (Huang et al. 2017). The k-turn structures of the snoRNPs appear to be a major locus for the control of assembly.

Thus, L7Ae binds to elements in the 5′-UTR in transcripts of multiple genes encoding proteins required in snoRNP assembly, including its own *l7ae* gene, altering the conformation of the RNA to modulate translation of the gene. These elements are genetic OFF switches that are mediated by a change in RNA conformation. They meet all the requirements for the definition of a riboswitch, except that most define a riboswitch as a motif in mRNA that responds to the binding of a small-molecule metabolite. Clearly a protein is not a small molecule, yet some authors consider the T-box element to be a riboswitch, despite the fact that its ligand is tRNA which is hardly a small metabolite. Whether or not this is considered to be a riboswitch is largely a question of definition, yet it conforms mechanistically to all the usual aspects of riboswitch function apart from the size of the ligand. The key aspect of the riboswitch mechanism in most cases is a ligand-induced change in RNA conformation, and that is what happens when L7Ae binds the k-turn. Yet however this is classified, the modulation of expression is mediated by protein-induced alteration of the RNA structure and represents an important mechanism of riboregulation.

## MATERIALS AND METHODS

### RNA synthesis and deprotection

RNA oligonucleotides were synthesized using the solid-phase phosphoramidite chemistry implemented on an ABI-394 DNA/RNA synthesizer. The ribonucleotide phosphoramidites, Pac-A-CE, Ac-C-CE, iPr-Pac-G-CE, and U-CE, with a *t*-BDMS protecting group on the 2′O, were obtained from Link Technologies. Fluorescein (Link Technologies) and Cy3 (GE Healthcare) were attached to the 5′ termini of the oligonucleotides as phosphoramidites in the final cycle of the synthesis, as required.

RNA oligonucleotides were deprotected in 25% ethanol/ammonia solution for 3 h at room temperature and evaporated to dryness. They were redissolved in 115 µL DMSO (Sigma-Aldrich) to which was added 60 µL triethylamine (Sigma-Aldrich) and 75 µL 1 M triethylamine trihydrofluoride (Sigma-Aldrich), and incubated at 65°C for 2.5 h to remove the *t*-BDMS protecting groups. Thereafter, samples were cooled on ice for 10 min and 250 µL RNA quenching buffer (Glen Research) was added to stop the reaction. The oligonucleotides were then desalted by application to NAP-10 columns (GE Healthcare).

### RNA purification and hybridization

Oligoribonucleotides were purified by gel electrophoresis under denaturing conditions, using 20% acrylamide: bis-acrylamide (19:1) (Scientific Laboratory Supplies) electrophoresed in 90 mM Tris-borate (pH 8.5), 10 mM EDTA (TBE buffer) containing 7 M urea at 25 W for ∼3 h. Nucleic acids were visualized by UV shadowing and bands corresponding to full-length products were excised and electroeluted into 140 µL 8 M ammonium acetate in TBE at 150 V at room temperature. The RNA was then precipitated with ethanol.

Fluorophore-labeled RNA was subjected to further purification by reversed-phase HPLC on a C18 column (ACE 10-300, Advanced Chromatography Technologies), using an acetonitrile gradient with an aqueous phase of 100 mM triethylammonium acetate (pH 7.0) (Fisher Scientific). Peak fractions were evaporated to dryness and resuspended in 120 µL ultrapure water.

For FRET experiments, duplex species were prepared by mixing equimolar quantities of oligoribonucleotides and annealing in 90 mM Tris-borate (pH 8.5), 10 mM EDTA, and 25 mM NaCl by slow cooling from 95°C to 4°C. Hybridized RNA was purified by gel electrophoresis under nondenaturing conditions in 12% acrylamide: bis-acrylamide (29:1), in TBE, 25 mM NaCl, with buffer recirculation. Electrophoresis was performed at 150 V at 4°C for ∼6 h. Bands containing duplex RNA were excised from the gel and electroeluted into 140 µL 8 M ammonium acetate in TBE at 100 V at 4°C, followed by ethanol precipitation and air-drying at 4°C.

### L7Ae expression and purification

*A. fulgidus* L7Ae cloned into a modified pET-Duet1 plasmid (Novagen) (Huang et al. 2011) was expressed as a hexahistidine fusion protein in *Escherichia coli* BL21-Gold (DE3) pLysS cells (Stratagene), and purified as reported previously (Huang and Lilley 2013).

### Steady-state FRET analysis of RNA folding

FRET experiments were performed using a duplex RNA species, terminally 5′-labeled with fluorescein on the bulged strand and Cy3 on the nonbulged strand, containing a central *l7Ae* 5′-UTR element k-turn sequence (Fig. 2). The strands used to prepare the duplex RNA species were (all sequences written 5′–3′): Flu-CCAGUCAGGGCCGAUGAAUGAGUUCAGG and Cy3-CCUGAACUCAUGAAGCCCUGACUGG; when hybridized together these generated a C-helix of 12 bp, and an NC-helix of 13 bp. The absorption spectrum was measured by resuspending the ethanol precipitated hybrid RNA in 120 µL 90 mM Tris-borate (pH 8.4) and recording the absorbance in a 50 mm path length cuvette using a NanoDrop 2000c spectrophotometer (Thermo Scientific). The spectrum was deconvoluted using corresponding RNA species labeled only with Cy3, and fluorophore absorption ratios calculated using a MATLAB program. Fluorescence spectra were recorded in 90 mM Tris-borate (pH 8.4) at 4°C using an SLM-Aminco 8100 fluorimeter. The spectra were corrected for lamp fluctuations and instrumental variations, and polarization artifacts were avoided by crossing excitation and emission polarizers at 54.7°. Values of FRET efficiency were measured using the acceptor normalization method (Clegg 1992) implemented in MATLAB. *E*_FRET_ as a function of L7Ae concentration was fitted to

(1) $$E_{\mathrm{FRET}}=E_{0}+\Delta E_{\mathrm{FRET}}\cdot \;\frac{(1+K_{A}P_{T}+K_{A}R_{T})-\sqrt{{(1+K_{A}P_{T}+K_{A}R_{T})}^{2}-4R_{T}K_{A}^{2}P_{T}}}{2R_{T}K_{A}},$$

where *E*_0_ is the initial FRET efficiency in the absence of added protein, *ΔE*_FRET_ is the full range of the change in FRET efficiency, *K*_*A*_ is the apparent association constant, and *P*_*T*_ and *R*_*T*_ are the total concentration of L7Ae and RNA, respectively.

### Crystallization, structure determination, and refinement

A solution of 0.6 mM RNA (19 nt) and 0.6 mM L7Ae in 5 mM Tris-HCl (pH 8.0), 100 mM NaCl, 10 mM MgCl_2_ was incubated for 1 min at 85°C. Crystals were grown by vapor diffusion using drops prepared by mixing 1.0 µL of the RNA–protein complex with 1 µL of a reservoir solution comprising 0.01 M Mg acetate, 0.05 M MES pH 5.6, and 2.5 M ammonium sulfate at 7°C. Crystals appeared after 5 d. They were transferred into reservoir solution containing 30% glycerol for ∼3 sec. The crystals were flash frozen by mounting in nylon loops and plunging into liquid nitrogen. The crystals were characterized in-house with a MicroMax HF007 copper rotating anode X-ray generator equipped with an ACTOR sample changer system and a Saturn 944HG+ CCD detector (Rigaku). Suitable samples were stored and subsequently used to measure full data sets on beamline I03 of Diamond Light Source (Harwell). Data were processed by XIA2 (Winter et al. 2018). The resolution cutoff for the data was determined by examining by CC1/2 and density map as described previously (Karplus and Diederichs 2012). The crystals had space group I422 and unit cell dimensions a = 142.1 Å, b = 142.1 Å, c = 166.8 Å. From crystal density considerations (Matthews 1968; Kantardjieff and Rupp 2003), one and a half RNA–protein complexes were expected to be present in the asymmetric unit.

The structure was determined by molecular replacement using the k-turn L7Ae complex model extracted from PDB 4BW0. The resulting electron density maps revealed the remaining RNA density and were built de novo on the basis of the difference map. Structural models were built in Coot (Emsley et al. 2010) and refined by Phenix (Adams et al. 2010). Omit maps were calculated using Phenix. Ramachandran analysis shows that 99.7% of amino acid residues are in the most favored and additionally allowed regions. Model geometry and the fit to electron-density maps were monitored with MOLPROBITY (Chen et al. 2010) and the validation tools in COOT. Atomic coordinates and structure factor amplitudes have been deposited with the PDB with accession code 6HCT.

### Analysis of L7Ae binding by in-line probing

In-line probing assays were performed as previously described (Soukup and Breaker 1999; Regulski and Breaker 2008). A total of 20 µL volumes of 100 pmol of 5′-Cy5-labeled RNA in 50 mM Tris-HCl (pH 8.3 at 20°C), 20 mM MgCl_2_, 100 mM KCl were supplemented with 2.5–10 µM L7Ae where required. RNA was folded by exposure to 85°C for 1 min followed by slow cooling to 20°C. Samples were then incubated at 20°C for 40 h, after which gel electrophoresis loading buffer containing 7 M urea, 10 mM EDTA was added. Partial alkaline digest was performed by incubation of 100 pmol of Cy5-labeled RNA in 20 µL 50 mM Na_2_CO_3_ buffer (pH 9.0 at 20°C) at 90°C for 10 min. The reaction was terminated by addition of gel loading buffer at 0°C. Each Cy5-labeled RNA was subjected to cleavage by RNase T1. 100 pmol of RNA was incubated with 5 U RNase T1 in 10 µL 25 mM sodium citrate buffer (pH 5.0 at 20°C) containing 5 M urea, 0.5 mM EDTA for 10 min at 55°C. The reaction was terminated by addition of gel loading buffer. Samples were electrophoresed in a 20% polyacrylamide gel containing 7 M urea. RNA was visualized by using a Typhoon FLA 9500 fluorimager (GE Healthcare).

## DATA DEPOSITION

Coordinates have been deposited in the Protein Data Bank with accession code 6HCT.

## SUPPLEMENTAL MATERIAL

Supplemental material is available for this article.

## Supplementary Material

Supplemental Material
