# Supplementary material for: The role of RNA structure in translational regulation by L7Ae protein in archaea
Source: RNA. 2019 Jan;25(1):60–9. doi: 10.1261/rna.068510.118 (PMC6298567; doi:10.1261/rna.068510.118)
Supplement: Supplemental Material [file supp_25_1_60__index.html]

The role of RNA structure in translational regulation by L7Ae protein in archaea — Supplemental Material 

# The role of RNA structure in translational regulation by L7Ae protein in archaea

## Supplemental Material

- Supplemental\_Information.pdf
