# Supplementary material for: The role of RNA structure in translational regulation by L7Ae protein in archaea
Source: RNA. 2019 Jan;25(1):60–9. doi: 10.1261/rna.068510.118 (PMC6298567; doi:10.1261/rna.068510.118)
Supplement: Supplemental Material [file supp_068510.118_Supplemental_Information.pdf]

The role of RNA structure in translational regulation by L7Ae protein in archaea

Lin Huang, Saira Ashraf and David M.J. Lilley

SUPPLEMENTARY INFORMATION

SUPPLEMENTARY FIGURES

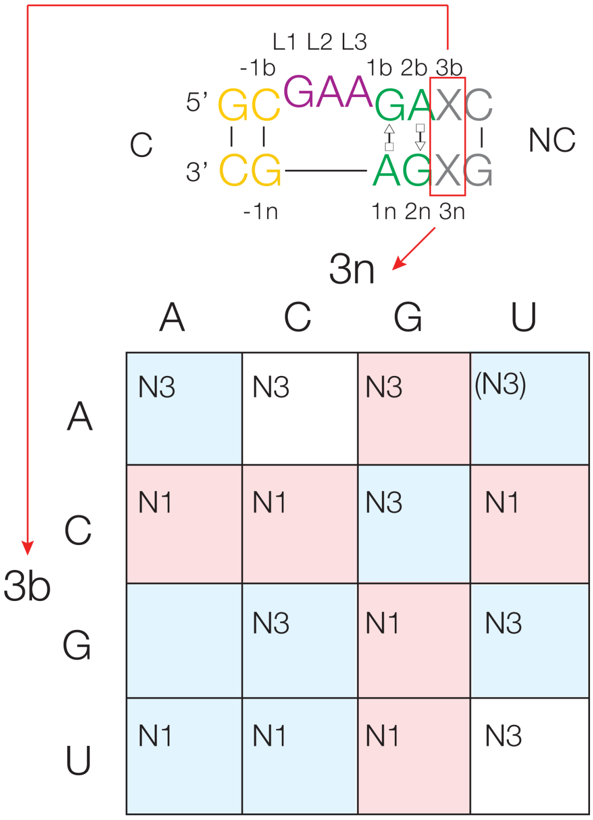

Figure S1. Summary of previous data showing the influence of the 3b:3n sequence on the folding and structure of *H. marismortui* Kt-7 (Huang et al., 2016, McPhee et al., 2014). Each square of the array corresponds to one of the 16 possible base pairs at the 3b:3n position, where the rows correspond to the 3b nucleotides and the columns are the 3n nucleotides. The color indicates the ability of a given sequence to fold into the kinked k-turn conformation in the presence of divalent metal ions alone (no protein). Red undergo folding, blue fail to fold in metal ions. The 3b:3n sequences also confer the conformation in terms of the hydrogen bond acceptor at A2b (donated by C-1n O2') i.e. whether an N3 or N1 structure is adopted. These are written into the squares where known. When these data were originally obtained the conformation of the k-turn structure with 3b:3n = A:U (top right, shown in parenthesis) was not known, but from bioinformatic data we deduced that it probably adopted the N3 conformation. This is confirmed in the present study.

Huang L, Wang J, Lilley DMJ. 2016 A critical base pair in k-turns determines the conformational class adopted, and correlates with biological function. *Nucleic acids res* **44**: 5390-5398

McPhee SA, Huang L, Lilley DMJ. 2014 A critical base pair in k-turns that confers folding characteristics and correlates with biological function. *Nature comm* **5**: 5127

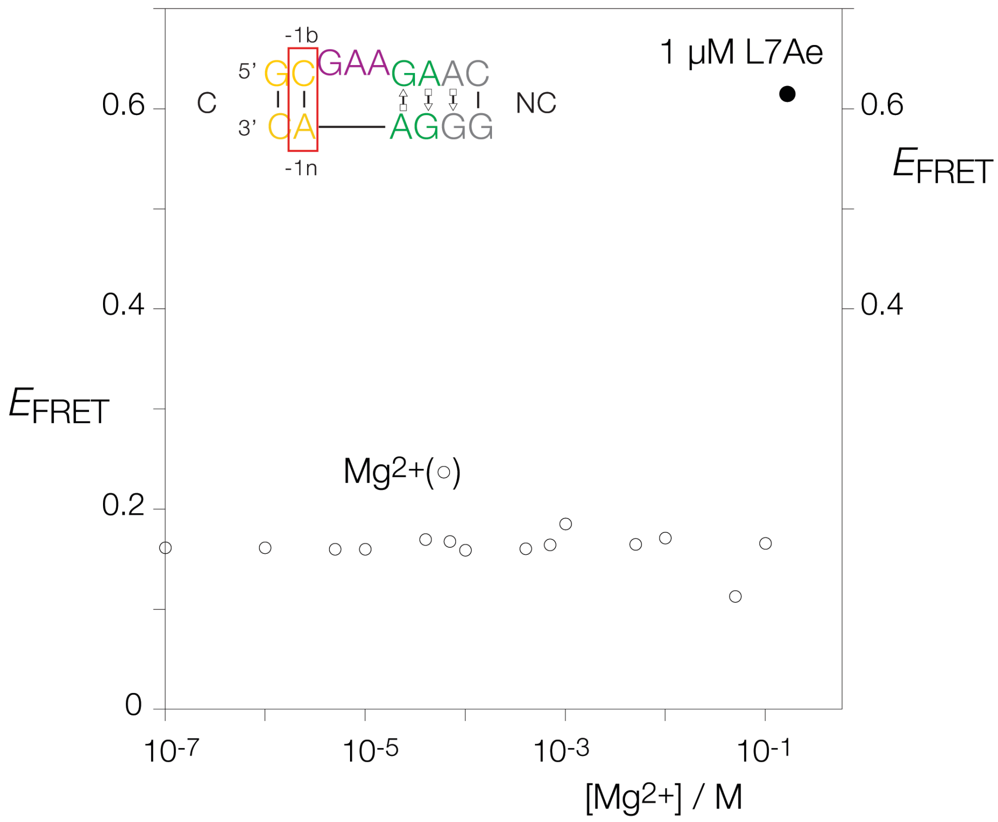

Figure S2. Folding of the Kt-7 k-turn with -1b:-1n sequence replaced by C:A. This is the sequence that occurs in the *A. fulgidus* l7ae URT putative k-turn. The variant Kt-7 sequence is shown inset, with the -1b:-1n sequence boxed. The sequence was placed in our normal Kt-7 environment used in FRET experiments. FRET efficiency (E_{FRET}) was measured as a function of Mg^{2+} ion concentration (open circles). After the final addition of $MgCl_2$ *Af*L7Ae was added to 1 μM concentration and E_{FRET} measured (filled circle). Note that the variant Kt-7 fails to undergo folding at any Mg^{2+} ion concentration, but addition of L7Ae results in complete folding (E_{FRET} = 0.615).

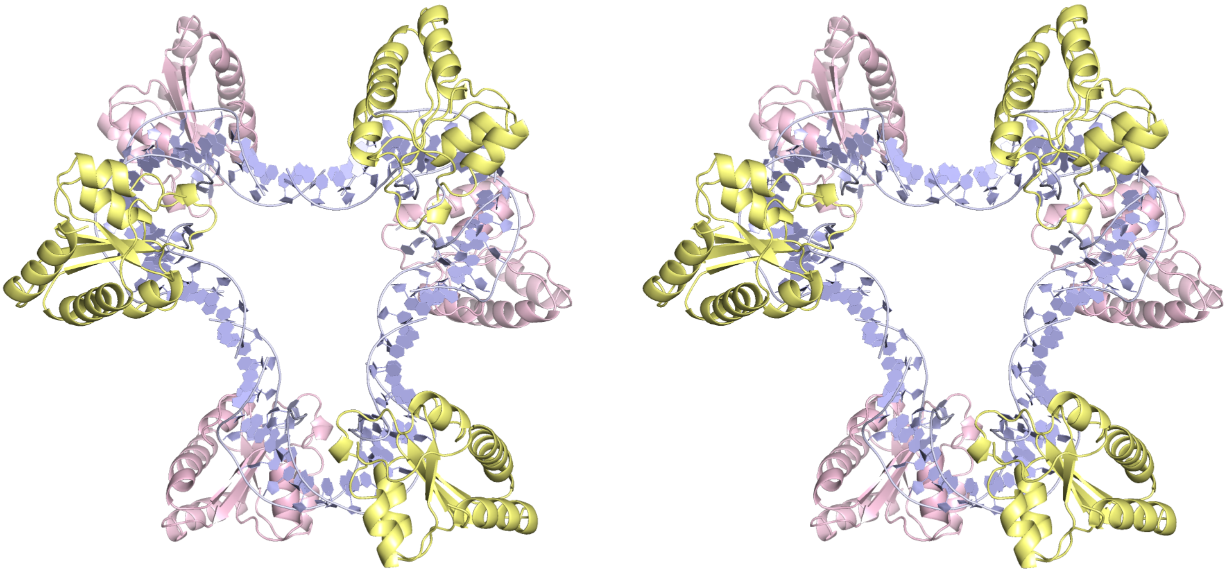

Figure S3. A triangular arrangement of *Af*L7Ae-k-turn RNA in the crystal lattice. Three double-k-turn RNA species adopt a triangular arrangement in the lattice with a three-fold rotational axis of symmetry. This comprises six k-turns and six bound *Af*L7Ae molecules. The asymmetric unit is one half of the triangle.

SUPPLEMENTARY TABLES

PDB ID	mother liquor	resolution	space group	beamline
6HCT	0.01 M Mg acetate<br>0.05 M MES (pH 5.6)<br>2.5 M $(NH_4)_2SO_4$	3.09 Å	I 422	Diamond IO3

Table S1. Summary of the RNA species, ligands and crystallization conditions used in these experiments, and the crystals obtained.

PDB	6HCT
**Data collection**	
Space group	I422
Cell dimensions	
a, *b*, *c* (Å)	142.1, 142.1, 166.8
α, β, γ (°)	90 90 90
Wavelength	0.9196
Resolution (Å)	83.40 – 3.09 (3.14 – 3.09)
R_{merge}	0.086 (0.914)
$I / \sigma I$	14.0 (2.1)
CC (1/2)	1.00 (0.38)
Completeness (%)	100.0 (100.0)
Redundancy	10.6 (10.5)
**Refinement**	
Resolution (Å)	70.07 – 3.09 (3.20 – 3.09)
No. reflections	15993 (1568)
R_{work} / R_{free}	0.187/ 0.236
No. atoms	
Macromolecules	3933
ligands	9
B-factors	
Macromolecules	112.32
ligands	108.90
Ramachandran favored (%)	95.36
Ramachandran allowed (%)	4.35
Ramachandran outliers (%)	0.29
Rotamer outliers (%)	0.00
R.m.s. deviations	
Bond lengths (Å)	0.009
Bond angles (°)	1.33

*Values in parentheses are for highest-resolution shell.

Table S2. Details of data collection and refinement statistics for the crystallographic data as deposited with the PDB.
